# Endoscopic submucosal dissection for severe fibrosis using a combined water pressure and circumferential-inversion method

**DOI:** 10.1055/a-2223-0405

**Published:** 2024-01-09

**Authors:** Hiroshi Takayama, Toshitatsu Takao, Douglas Motomura, Hitomi Hori, Yoshinori Morita, Takashi Toyonaga, Yuzo Kodama

**Affiliations:** 1 592910 Division of Gastroenterology, Kobe University Graduate School of Medicine Department of Internal Medicine, Kobe, Japan; 2 8166 Department of Gastroenterology, The University of British Columbia, Vancouver, Canada; 3 Department of Gastroenterology, Kobe University Hospital International Clinical Cancer Research Center, Kobe, Japan

Use of the water pressure method during endoscopic submucosal dissection (ESD) has been reported to shorten procedure times for colorectal lesions with fibrosis 1 2 ; however, ESD for cases with severe fibrosis remains extremely challenging 3 . We recently reported a novel traction method called the circumferential-inversion method (CIM), which involves inverting the lesion circumferentially 4 . In this report, we describe the effectiveness of ESD using a novel approach that combines the water pressure method and CIM (WP-CIM) for lesions with severe fibrosis ( Video 1 ).

Endoscopic submucosal dissection is performed using a novel approach that combines the water pressure method and the circumferential-inversion method for a lesion on a scar with severe fibrosis.Video 1

The case involved a 69-year-old woman with a 30-mm 0-IIa tumor on the scar created by a previous endoscopic submucosal resection in the sigmoid colon ( Fig. 1 **a** ). Local injection at the scar site did not result in any elevation ( Fig. 1 **b** ). We attempted ESD using the water pressure method; however, it was challenging to approach the submucosal layer at the scar site ( Fig. 1 **c,d** ). After performing a complete circumferential incision and trimming, we grasped the specimen by applying an orthodontic rubber band (inner diameter, 8 mm) and clips (SureClip 8 mm; Micro-Tech, Nanjing, China) from five directions ( Fig. 2 **a** ). The combination of the water pressure method and CIM enabled us to access the submucosal layer effectively ( Fig. 2 **b** ). Further dissection from the left and right sides allowed us to recognize the dissection line at the scar site ( Fig. 2 **c** ). The dissection was completed without any complications, resulting in an R0 resection ( Fig. 2 **d** ).

**Fig. 1:**
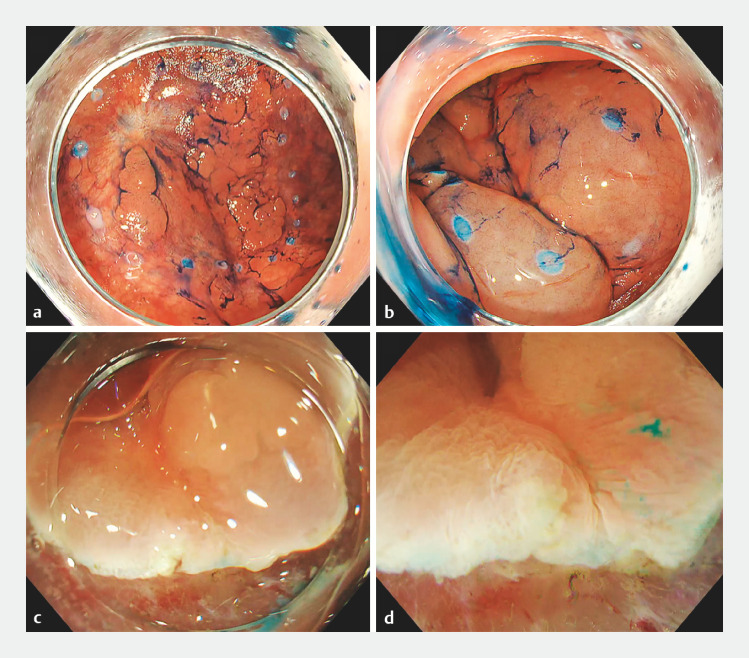
Endoscopic views during endoscopic submucosal dissection using the water pressure method showing: **a** a 30mm 0-IIa tumor on the scar created by endoscopic submucosal resection in the sigmoid colon; **b** lack of elevation following local injection at the scar site; **c** severe fibrosis at the scar site that made it challenging to approach the submucosal layer; **d** the water pressure method being used, but it remained challenging to approach the submucosal layer.

**Fig. 2:**
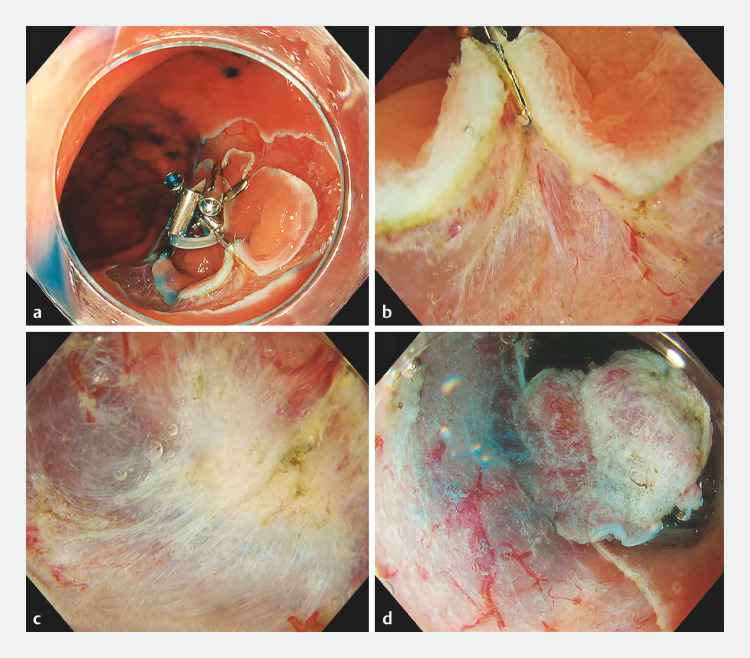
Endoscopic views during endoscopic submucosal dissection (ESD) using a novel approach that combines the water pressure method and the circumferential-inversion method (CIM; WP-CIM) showing: **a** the specimen grasped from five directions with an orthodontic rubber band and clips after complete circumferential incision and trimming had been performed; **b** WP-CIM being used, which allowed effective access to the submucosal layer; **c** further dissection from the left and right sides allowing recognition of the dissection line at the scar site; **d** R0 resection completed without complications using CIM-enhanced buoyancy and the water pressure effect.

Because CIM is inversion traction, it enhances the effectiveness of the water pressure method in an airless environment. Additionally, CIM improves the visibility of the dissection line at the scar site by promoting dissection not only from the front but also from the left and right sides. We propose that WP-CIM facilitates ESD for lesions with severe fibrosis.

Endoscopy_UCTN_Code_TTT_1AO_2AG

## Notes

Endoscopy E-Videos https://eref.thieme.de/e-videos
*E-Videos* is an open access online section of the journal *Endoscopy* , reporting on interesting cases and new techniques in gastroenterological endoscopy. All papers include a high-quality video and are published with a Creative Commons CC-BY license. Endoscopy E-Videos qualify for HINARI discounts and waivers and eligibility is automatically checked during the submission process. We grant 100% waivers to articles whose corresponding authors are based in Group A countries and 50% waivers to those who are based in Group B countries as classified by Research4Life (see: https://www.research4life.org/access/eligibility/ ). This section has its own submission website at https://mc.manuscriptcentral.com/e-videos .
